# Adaptive Darwinian off-target resistance mechanisms to selective RET inhibition in RET driven cancer

**DOI:** 10.1038/s41698-024-00563-4

**Published:** 2024-03-04

**Authors:** Vivek Subbiah, Mohamed A. Gouda, J. Bryan Iorgulescu, Ramona Dadu, Keyur Patel, Steven Sherman, Maria Cabanillas, Mimi Hu, Luz E. Castellanos, Behrang Amini, Funda Meric-Bernstam, Tao Shen, Jie Wu

**Affiliations:** 1 Department of Investigational Cancer Therapeutics, The University of Texas MD Anderson Cancer Center, Houston, TX USA; 2 Molecular Diagnostics Laboratory, Department of Hematopathology, Division of Pathology and Laboratory Medicine, The University of Texas MD Anderson Cancer Center, Houston, TX USA; 3 Department of Endocrine Neoplasia, The University of Texas MD Anderson Cancer Center, Houston, TX USA; 4 Peggy and Charles Stephenson Cancer Center and Department of Pathology, University of Oklahoma Health Sciences Center, Oklahoma City, OK USA; 5 Present Address: Sarah Cannon Research Institute, Nashville, TN USA

**Keywords:** Cancer therapeutic resistance, Cancer therapeutic resistance

## Abstract

Patients treated with RET protein tyrosine kinase inhibitors (TKIs) selpercatinib or pralsetinib develop RET TKI resistance by secondary RET mutations or alterative oncogenes, of which alterative oncogenes pose a greater challenge for disease management because of multiple potential mechanisms and the unclear tolerability of drug combinations. A patient with metastatic medullary thyroid carcinoma (MTC) harboring a RET activation loop D898_E901del mutation was treated with selpercatinib. Molecular alterations were monitored with tissue biopsies and cfDNA during the treatment. The selpercatinib-responsive MTC progressed with an acquired ETV6::NTRK3 fusion, which was controlled by selpercatinib plus the NTRK inhibitor larotrectinib. Subsequently, tumor progressed with an acquired EML4::ALK fusion. Combination of selpercatinib with the dual NTRK/ALK inhibitor entrectinib reduced the tumor burden, which was followed by appearance of NTRK3 solvent-front G623R mutation. Preclinical experiments validated selpercatinib plus larotrectinib or entrectinib inhibited RET/NTRK3 dependent cells, whereas selpercatinib plus entrectinib was necessary to inhibit cells with RET/NTRK3/ALK triple alterations or a mixture of cell population carrying these genetic alterations. Thus, RET-altered MTC adapted to selpercatinib and larotrectinib with acquisition of ETV6::NTRK3 and EML4::ALK oncogenes can be managed by combination of selpercatinib and entrectinib providing proof-of-concept of urgency of incorporating molecular profiling in real-time and personalized N-of-1 care transcending one-size-fits-all approach.

## Introduction

Genomic instability is one of the hallmarks of cancer that enables the survival of malignant tumors^1,2^. The mutational landscape of tumors evolves over time to evade destruction of cancer cells by therapeutic intervention or immune system^3^. Such evolution is a common reason for secondary resistance that occurs for almost all new therapies despite initial outstanding responses^3,4^. In the past, treatment paradigms relied on changing the drug used once tumor progression was observed. However, this approach was predominantly conceived during the era of chemotherapy. At that time, it seemed reasonable because the regimens used were not specifically aimed at oncogenic drivers in cancer cells, but instead affected all dividing cells in the human body. Moreover, physicians lacked knowledge about the molecular basis that occurred in a particular patient after tumor progression. In the era of precision oncology, significant technological advancements, such as next-generation sequencing and the adoption of more accessible methods like liquid biopsy, have revolutionized cancer treatment. These breakthroughs now allow for timely detection of changes in molecular profile of cancer induced by a treatment. As a result, acquired resistance mechanisms caused by these alterations may be promptly identified and addressed, leading to more effective personalized therapy^4–7^.

Whereas RET-selective protein tyrosine kinase inhibitors selpercatinib and pralsetinib rendered high rates of responses in RET-altered cancers, less than 10% of patients achieved a complete response^8–16^. The presence of residual tumors in most patients with RET-altered cancer after selpercatinib or pralsetinib treatment requires long-term disease management. Because genomic instability, residual tumors will adapt to these TKIs and evolve to drug resistance through secondary “on-target” *RET* mutations or acquisition of alterative “off-target” oncogenes^17–23^. On-target mechanism of resistance to selpercatinib occurs most often in the RET G810 solvent-front site. Several next-generation of RET inhibitors are in preclinical or clinical development to overcome RET G810 solvent-front mutations^15,16,24^. However, although RET solvent-front mutation is a well-defined mechanism of selpercatinib and pralsetinib resistance that can be addressed with new RET TKIs, the rates of selpercatinib- and pralsetinib-resistant RET mutations are believed to be relatively low and is reported to be around 10% in a small study cohort^19^. In comparison, selpercatinib- or pralsetinib-resistance caused by alterative oncogenes is a more complex problem due to the complexity of alternative oncogenic drivers and the presumptive predominance of off-target mechanisms in acquired resistance to selpercatinib and pralsetinib^21–23^.

Previously, we reported a selpercatinib-treated *KIF5B::RET*-positive lung cancer patient whose disease progressed with acquired *KHDBS1::NTRK3* fusion^23^. The selpercatinib-resistant *KHDBS1::NTRK3* fusion was discovered after the patient had succumbed to the progressive disease, missing the opportunity of timely intervention. In this report, we present a patient with MTC harboring a previously uncharacterized RET activation loop deletion mutation (p.D898_E901del), who had a nice response to selpercatinib but developed target-bypass secondary, tertiary and quaternary genetic lesions as resistance mechanisms after 24 months of the RET-targeted therapy. We describe adaptive therapeutic intervention using combination of targeted agents that target patient’s evolving mechanisms of resistance. This exemplifies a practical approach to tackle the intricacies of cancer treatment during the targeted therapy era^4,25^. It demonstrates that customized combination therapies can effectively address the complexity of evolving resistance mechanisms, leading to prolonged tumor control in long-term disease management.

## Results

### Characterization of p.D898_E901del deletion mutant in the RET kinase activation loop

**Fig. 1:**
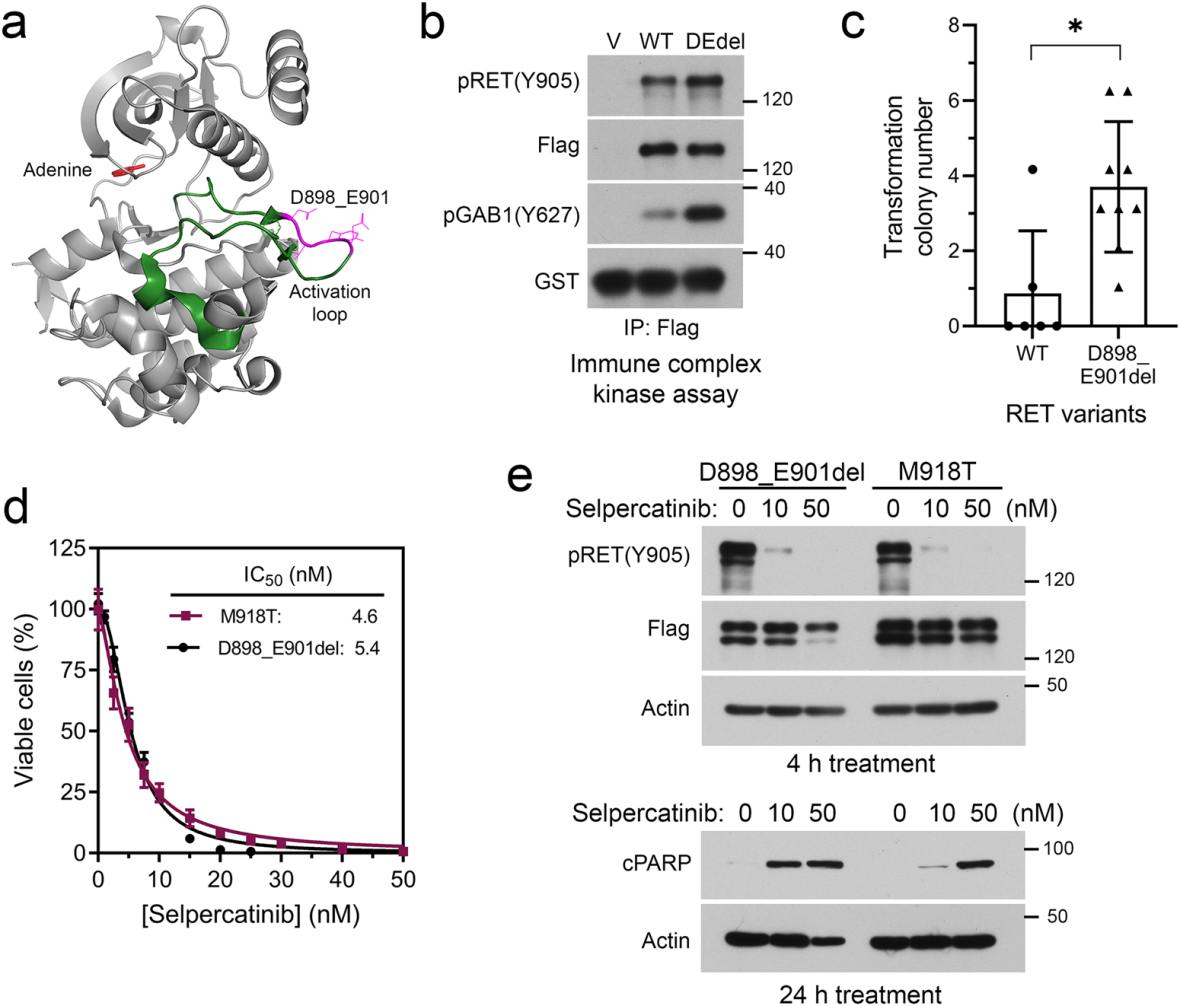
Characterization of the RET activation loop deletion mutant. **a** A crystal structure of the RET kinase domain (PDB id: 6NJA) showing activation loop (green); amino acids D898_E901 (magenta); and the adenine group of bound ATP (red). **b** Immune complex kinase assay comparing the kinase activity of wildtype RET and the RET(D898_E901del) mutant. **c** Cytokine-independent transformation activity of wildtype RET and the RET(D898_E901del) mutant. Each data point represents the number of cytokine-independent colonies in a 96-well plate. The data were from two (wildtype) or three (D898_E901del) independent experiments. *p < 0.05. **d** Selpercatinib IC_50_ determination of BaF3/RET(D898_E901del) and BaF3/RET(M918T) cells. Data were from two experiments performed in triplicates. **e** Cells were treated with indicated concentrations of selpercatinib. Cell lysates were analyzed by immunoblotting with indicated antibodies. GST, glutathione S-transferase, which was used as a marker for the recombinant GST-GAB1CT protein. cPARP, cleaved poly(ADP-ribose) Polymerase (PARP), which is a marker of apoptotic cells.

Tumor tissue and plasma cfDNA sequencing revealed a rare somatic in-frame *RET* deletion [NM_020975.6(*RET*):c.2694_2705del] in the MTC patient reported here. The c.2694_2705del results in a deletion of four amino acid residues D898–E901 in the activation loop of the RET kinase domain (Fig. 1a). To determine if the D898–E901 deletion affects RET tyrosine kinase activity, we performed an immune complex kinase assay^26^. As shown in Fig. 1b, the RET(D898_E901del) mutant displayed elevated autophosphorylation and phosphorylation of RET kinase substrate protein GAB1 than the wildtype RET. In a BaF3 cell cytokine-independence transformation assay, RET(D898_E901del) has significantly higher transformation activity than the wildtype RET (Fig. 1c). These results demonstrated that the previously uncharacterized RET activation loop deletion mutant has constitutively active kinase activity. To assess if RET(D898_E901del) is sensitive to selpercatinib, we compared the responses of BaF3/RET(D989_E901del) cells and BaF3/RET(M918T) cells to the drug. M918T is the most common somatic RET mutation in MTC. As shown in Fig. 1d, selpercatinib had similar IC_50s_ of 5.4 nM and 4.6 nM for BaF3/RET(D898_E901del) cells and BaF3/RET(M918T) cells, respectively. Immunoblotting assay confirmed inhibition of RET(D898_E901del) and RET(M918T) by selpercatinib with a similar potency.

### Case presentation

A female patient in her 40 s presented with self-palpated breast masses and thyroid nodules. Biopsy revealed medullary thyroid carcinoma (MTC) and tumor markers showed elevated CEA (141.8 ng/mL) and calcitonin (568 pg/mL). Initial staging workup suggested the presence of multi-focal liver metastasis, enlarged abdominal lymph nodes, multiple pulmonary lymph nodes, and several osseous lesions. Upon multidisciplinary discussion, patient underwent total thyroidectomy and central neck dissection. During surgery, she also had lumpectomy of the two breast masses. Pathology of surgical specimen showed metastatic medullary thyroid carcinoma with foci of micropapillary thyroid cancer. Tissue DNA sequencing – using an MD Anderson paired tumor-normal next-generation sequencing assay that analyzed 146 cancer-related genes-suggested the presence of short in-frame deletion of *RET* coding sequence that resulted in four amino acid deletion [NM_020975.6(RET):c.2094_2705del p.D898_E901del] in the RET kinase activation loop in both tissue and plasma samples. Variant allele frequency (VAF) of *RET* p.D898_E901del in plasma was 12% (using an MD Anderson cell-free DNA liquid biopsy NGS assay that analyzed 70 cancer-related genes).

**Fig. 2:**
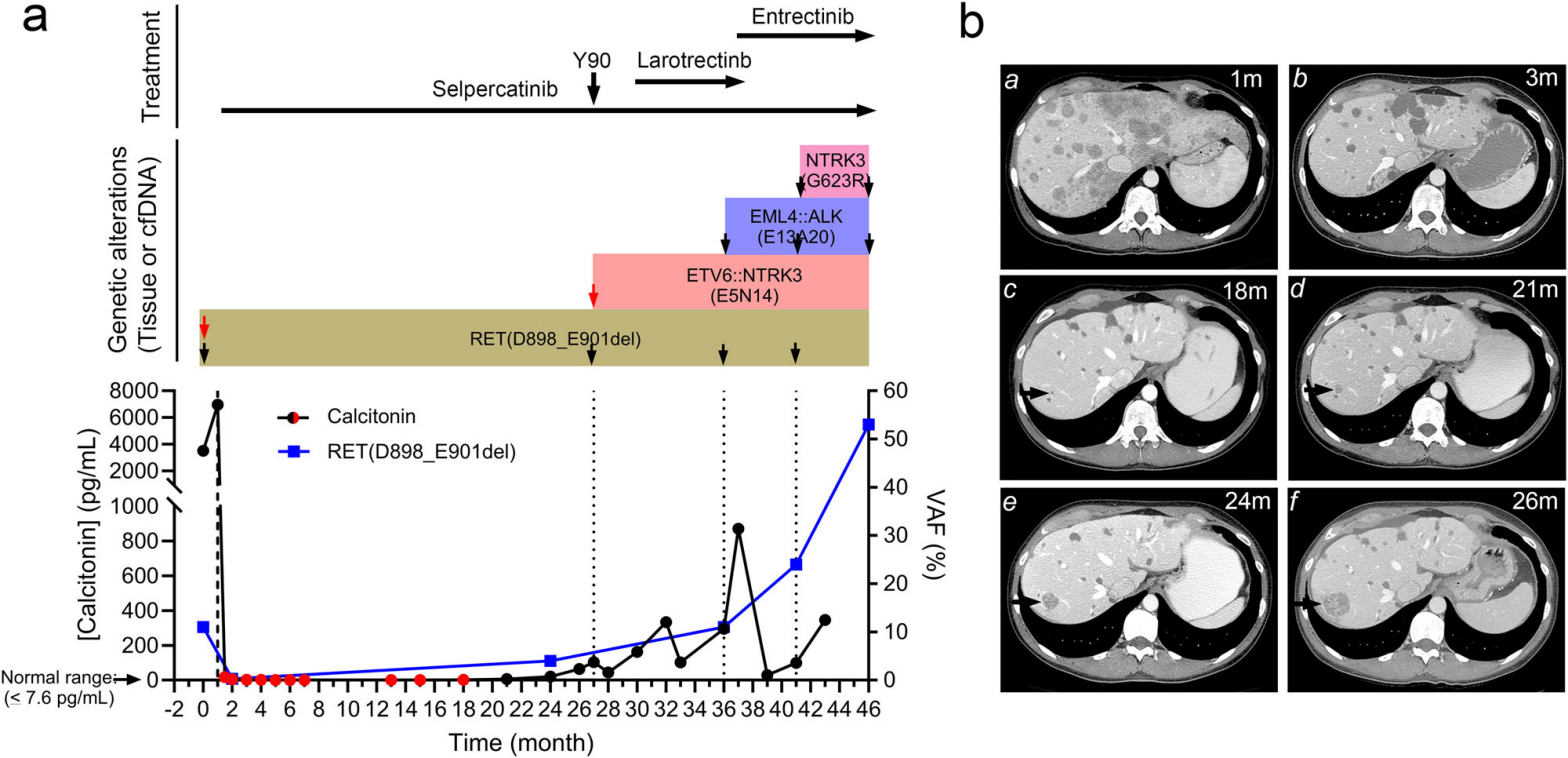
Drug-driven evolution of *RET*-mutant metastatic MTC. **a** Patient’s calcitonin levels, variant allele frequency (VAF) of the *RET* p.D898_E901del mutation in cfDNA assay, the time points when genetic alterations were identified in samples from tissue biopsy (red arrows) or lipid biopsy (black arrows), and the treatment history. Red circle points of calcitonin were those in the normal range. **b** Contrast-enhanced computed tomography (CT) imaging of the abdomen over time: a, baseline scan shows innumerable hepatic metastases; b, first follow-up scan shows significant positive response to therapy with decrease in size and enhancement of multiple lesions; c, CT shows appearance of a tiny new lesion (arrow). d–f, the lesion continues to enlarge on subsequent scans and was first noted on e and progression confirmed on f.

The patient was referred for treatment as part of clinical trial investigating the RET inhibitor, selpercatinib. The phase I/II trial (LIBRETTO-001; NCT03157128) included patients with advanced solid tumors and RET alterations. The patient provided written informed consent before enrollment. Imaging studies (MRI of the brain and spine; computed tomography of the neck, chest, abdomen, and pelvis) were performed every 8 weeks. The response was assessed according to RECIST, version 1.1. The patient started treatment with selpercatinib 160 mg twice daily one month after surgery and experienced clinical response within the first month of therapy, with resolution of baseline diarrhea and nausea, and appetite improvement. Consistent with resolution of diarrhea, calcitonin levels decreased from pre-treatment level of 6960 pg/mL to a normal level of 4.3 pg/mL within 1 month of treatment initiation (Fig. 2a). Similarly, CEA levels normalized from pre-treatment level of 708.9 pg/mL to 1.1 pg/mL after 1 month of treatment. Follow up imaging showed partial response of liver lesions and abdominal lymph nodes (-43% per RECIST 1.1) which was maintained for 24 months (best response was partial response -65% per RECIST 1.1).

While on therapy, follow up liquid biopsy after two years showed a decrease of *RET* p.D898_E901del VAF to 4% (from pretreatment VAF of 12%). However, after 24 months of treatment initiation, there was progression in the liver lesions (Fig. 2a, b). The patient underwent protocol-allowed local therapy with Yttrium-90 Microspheres radioembolization to control one of the larger liver masses that was progressing while continuing selpercatinib. A tissue biopsy of the progressing liver lesion showed an emerging *ETV6::NTRK3* (E5N14) fusion in addition to the *RET* p.D898_E901del (Fig. 2a and Table 1; using the aforementioned DNA tissue sequencing assay combined with an MD Anderson RNA-based NGS assay that detects fusions involving 51 genes). Histopathology was suggestive of metastatic carcinoma morphologically compatible with patient’s known medullary thyroid carcinoma. Post procedure, her calcitonin decreased, and immediate scans showed a response. However, eventually, metastatic lesions continued to grow, and the calcitonin and the CEA started rising again (Fig. 2a, b). Given the presence of *RET* alteration and new *NTRK3* fusion and after extensive discussion with patient, patient was removed off the LIBRETTO-001 clinical trial. Since selpercatinib received regulatory approval in the interim, we designed a customized regimen for the patient with the RET inhibitor, selpercatinib, for the *RET* p.D898_E901 deletion in combination with NTRK inhibitor, larotrectinib, for the *NTRK* fusion kinase. This was done step wise, calcitonin was rising with selpercatinib, so selpercatinib was stopped and larotrectinib was started, calcitonin continued to increase, then selpercatinib was added to larotrectinib. Selpercatinib was started at 80 mg po BID and larotrectinib was started at 50 mg po BID. Given the tolerance, selpercatinib was escalated to 160 mg po BID in 2 weeks and larotrectinib was escalated to 100 mg po BID. Side effects included grade 1 fatigue, grade 1 liver function test elevation, grade 1 dry mouth and, grade 1 peripheral neuropathy.

**Table 1:** Molecular alterations identified over time in an advanced MTC patient

Timepoint	Sequencing	Sample	*RET* p.D898_E901del VAF, %	*NTRK3* p.G623R VAF, %		*EML4::ALK* fusion	*ETV6::NTRK3* fusion
Month -1	Tissue	Primary tumor	74		<5	n/a	n/a
Month 0	cfDNA	Plasma	11		<0.3	Not detected	n/a
Month 2	cfDNA	Plasma	<0.3		<0.3	Not detected	n/a
Month 24	cfDNA	Plasma	4		<0.3	Detected^a^	n/a
Month 27	Tissue	Liver metastasis	38		<5	Not detected	Detected
Month 36	cfDNA	Plasma	11		<0.3	Detected	n/a
Month 41	cfDNA	Plasma	24		8	Detected	n/a
Month 46	cfDNA	Plasma	53		14	Detected	n/a

Timepoint is calculated from date of diagnosis

n/a: Assay did not include fusion detection or coverage of breakpoints. Of note, no other sequencing timepoints covered the *ETV6::NTRK3* fusion.

Fluctuations in mutations’ cfDNA VAF may be related to clonal evolution, changes in tumor burden, and/or other phenomena. The validated limit of detection was 5% for tissue sequencing and 0.3% for cfDNA sequencing assays.

^a^Only 1 read of the reverse fusion transcript was detected, which was only considered positive when the fusion was confirmed on cfDNA assays.

Calcitonin levels declined (Fig. 2a) and imaging studies showed a response in the liver lesions. Patient’s pain and fatigue were also better after the two-drug regimen combination. However, in seven months, calcitonin started rising and patient again started to feel pain. CT scan showed disease progression and a liquid biopsy revealed emergence of an *EML4::ALK* fusion in addition to *RET* p.D989_E901del whose VAF increased to 11%. Given *RET* deletion and *ALK* fusion in the context of *NTRK* fusion, the regimen was further personalized to selpercatinib and entrectinib. Entrectinib is a selective inhibitor of NTRK, ROS1, and ALK tyrosine kinases^27,28^. Therefore, selpercatinib was once again re-started at 80 mg po BID and treatment with ALK-TRK-ROS1 inhibitor entrectinib was initiated at 200 mg once daily. Selpercatinib was dose escalated to 160 mg po BID and entrectinib was escalated to 400 mg daily. Grade 1 memory issues, grade 1–2 fatigue, grade 1 visual disturbance, and grade 1 diarrhea were the side effects that were initially observed with the combination regimen. The patient, however, developed grade 2 dry eyes and dry mouth so the dose was de-escalated to 200 mg; but later re-escalated with the aid of artificial tears, xylitol and supportive care back to 400 mg. Calcitonin started decreasing after initiating the new combination regimen (decreased from 871 pg/mL to 28.6 pg/mL at two months) with her pain also resolving (Fig. 2a). A re-staging scan showed partial response and the patient was clinically stable. In later months, with mild calcitonin elevation in the context of stable scans, a liquid biopsy was performed again, which showed the emergence of an *NTRK3* p.G623R resistance mutation (VAF 8%) in addition to the persistent *EML4::ALK* and *RET* p.D898_E901del (VAF 24%). Accordingly, the patient was continued on treatment with selpercatinib and entrectinib for 9 months; but the patient eventually experienced disease progression including the development of brain metastatic disease. Liquid biopsy around time of progression showed *NTRK3* p.G623R (VAF 14%), *RET* p.D898_E901del (VAF 53%), *EML4::ALK*, and *CCNE1* gain. She received treatment with cabozantinib and everolimus but she unfortunately passed away few weeks later.

### Mono- and combinational-treatments on RET(D898_E901del), *ETV6::NTRK3*, and *EML4::ALK*-dependent cells

**Fig. 3:**
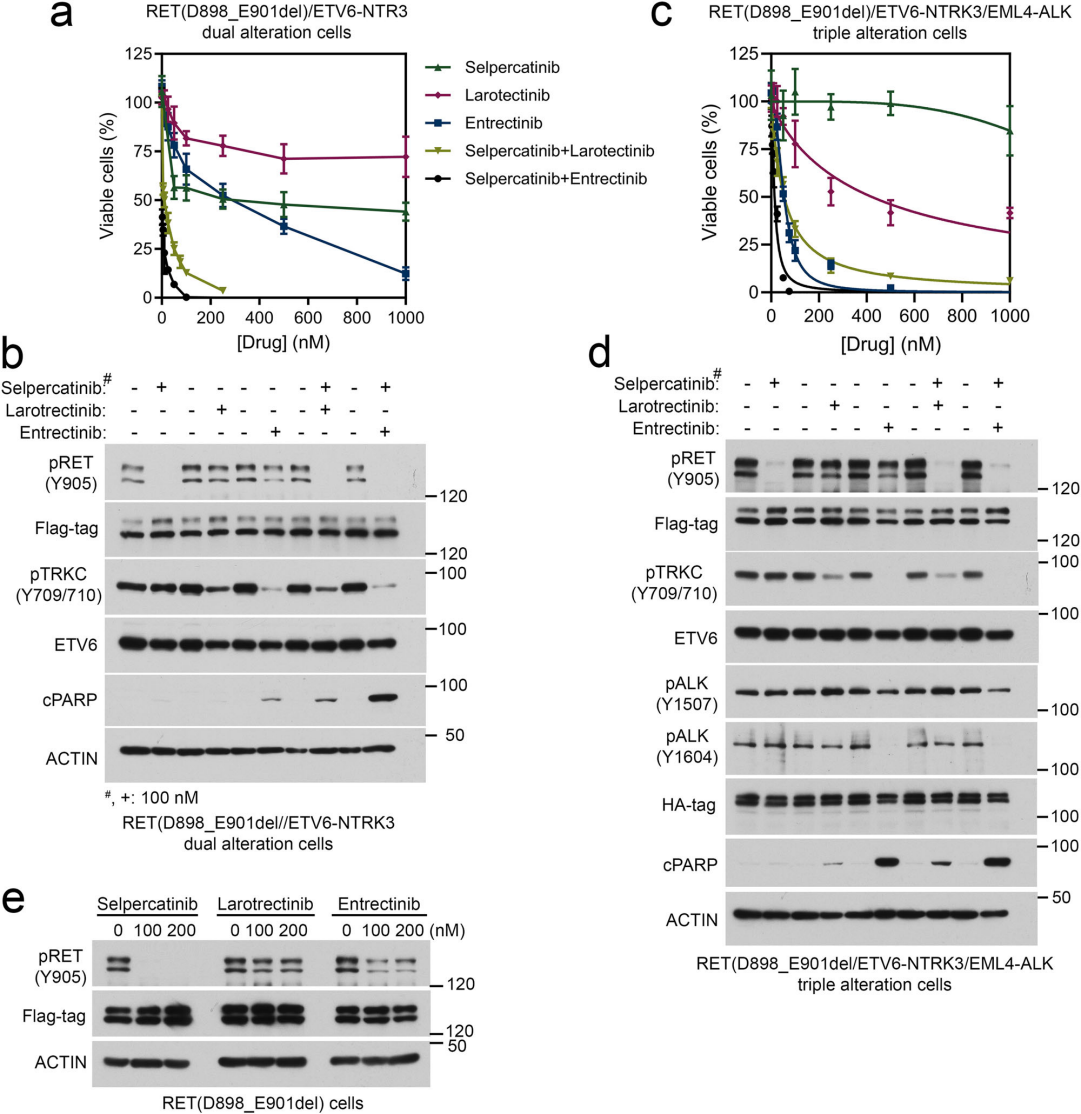
Sensitivities of cells containing two or three oncogenic kinases to selpercatinib, larotrectinib, entrectinib, and combination treatments. BaF3/RET(D898_E901del)/*ETV6::NTRK3* cells (**a**, **b**), or BaF3/RET(D898_E901del)/*ETV6::NTRK3*/*EML4::ALK* cells (**c**, **d**) were cultured in 96-well plates and treated with indicated drugs for three days, and viable cells were measured using CellTiter-Glo reagent. Response curves were shown (**a**, **c**). **b**, **d**, **e**. Cells were treated with indicated drugs (100 nM, or as indicated) for 4 h (kinase inhibition analyses) or 24 h (apoptosis analyses). Cell lysates were analyzed by immunoblotting with indicated antibodies.

To test whether cells harboring *RET* p.D898_E901del, *ETV6::NTRK3*, and *EML4::ALK* kinases are sensitive to selpercatinib, larotrectinib, and entrectinib, and their combinations, we evaluated the activities of these drugs in BaF3/RET(D898_E901del)/*ETV6::NTRK3* cells, BaF3/RET(D898_E901del)/*ETV6::NTRK3*/*EML4::ALK* cells, and a mixture of BaF3/RET(D898_E901del)/*ETV6::NTRK3* and BaF3/*EML4::ALK* cells. Selpercatinib, larotrectinib, or entrectinib alone had > 200 nM IC_50_s for the double RET(D898_E901del)/*ETV6::NTRK3* alterations cells, and could not completely suppress cell growth at 1000 nM. Combination of 100 nM selpercatinib with larotrectinib or entrectinib reduced the IC_50_ to 10 and 1.8 nM, respectively, in BaF3/RET(D898_E901del)/*ETV6::NTRK3* cells (Fig. 3a), demonstrating that combination of the RET inhibitor with a NTRK inhibitor was required to effectively control the cells containing both RET and NTRK3 oncogenes. Immunoblotting showed that only a combination of selpercatinib with larotrectinib or with entrectinib could inhibit both RET(D898_E901del) and *ETV6::NTRK3* kinase activities in these cells (Fig. 3b).

**Fig. 4:**
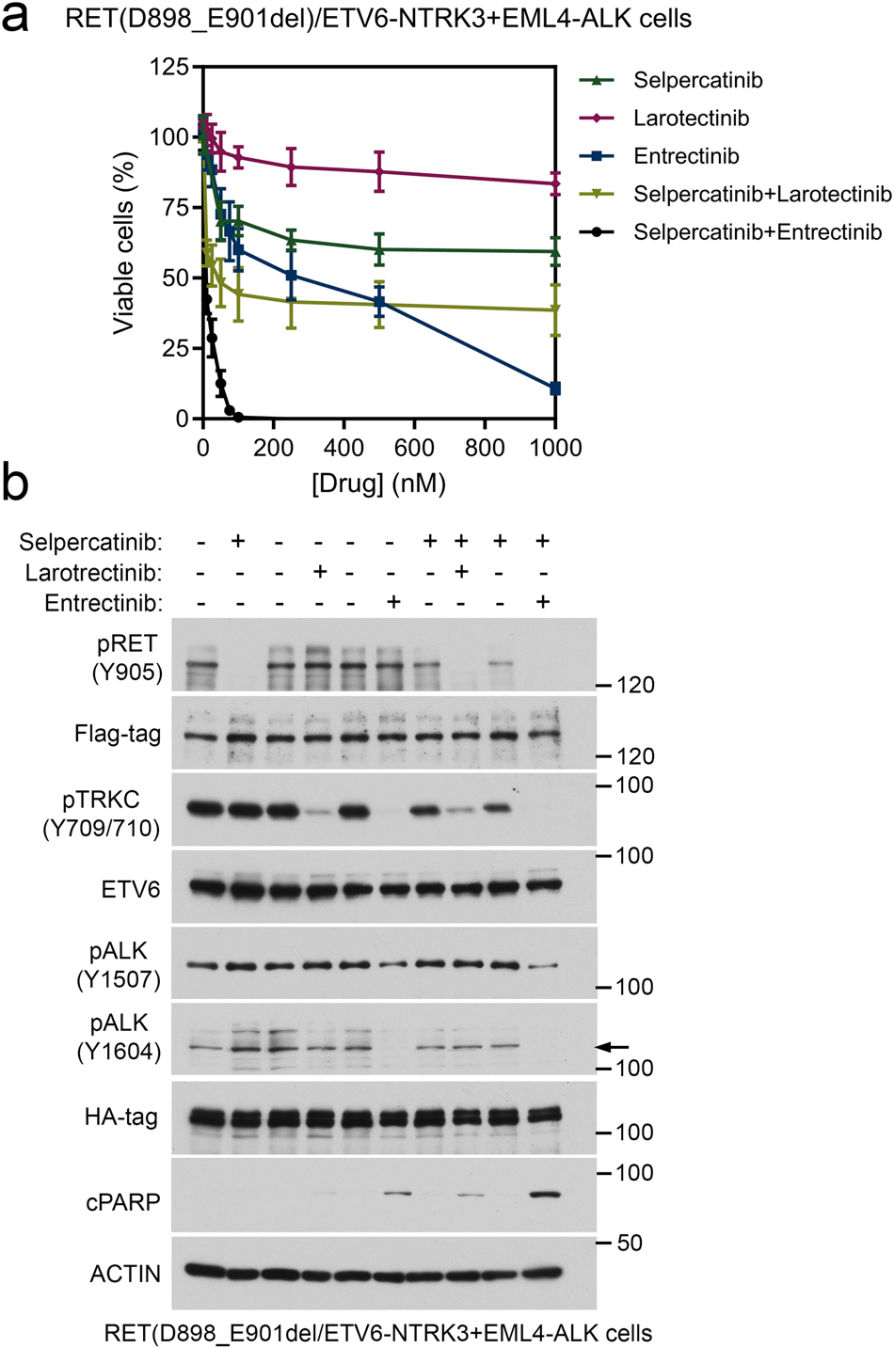
Sensitivities of a mixture population of BaF3/RET(D898_E901del)/ETV6-NTRK3 and BaF3/EML4-ALK cells to selpercatinib, larotrectinib, entrectinib, and combination treatments. **a** an equal number of two cell lines were mixed, cultured in 96-well plates and treated with indicated drugs for three days, and viable cells were measured using CellTiter-Glo reagent. **a**. Drug response curves. **b** Cells were treated with indicated drugs for 4 h (kinase inhibition analyses) or 24 h (apoptosis analyses). Cell lysates were analyzed by immunoblotting with indicated antibodies.

BaF3 cells containing triple RET(D898_E901del), *ETV6::NTRK3*, and *EML4::ALK* alterations, or a mixture of BaF3/RET(D898_E901del)/*ETV6::NTRK3* and BaF3/*EML4::ALK* cells were used to assess the combination treatment (Fig. 3c, d; Fig. 4, and Table 2.). In the RET/NTRK3/ALK3 triple alteration cells, combination of selpercatinib and entrectinib had the lowest IC50 (14.19 nM) and could completely suppress these cells at 75 nM (Fig. 3c and Table 2). While entrectinib alone or the combination of selpercatinib and larotrectinib had a modest 3.8-fold higher IC50s in the triple alteration cells, 1000 nM entrectinib was required to completely inhibit these cells and the selpercatinib/larotrectinib combination could not completely inhibit these cells at 1000 nM (Fig. 3c and Table 2). These responses are more apparent in the experiment a mixture of cell population (Fig. 4 and Table 2). Immunoblotting analysis showed that the combination of selpercatinib with entrectinib was the most effective in inhibition of all three tyrosine kinases (Fig. 3d). Moreover, entrectinib, but not larotrectinib, displayed partial RET kinase inhibition (Fig. 3d, e).

**Table 2:** IC_50_s and maximal inhibition

Treatment	BaF3 derived cell line					
	RET(898_901del)/ETV6-NTRK3		RET(898_901del)/ETV6-NTRK3/EML4-ALK		RET(898_901del)/ETV6-NTRK3 + EML4-ALK	
	IC_50_ (nM)	^a^Maximal inhibition (%)	IC_50_ (nM)	Maximal inhibition (%)^a^	IC_50_ (nM)	Maximal inhibition (%)^a^
Selpercatinib	290.6	55.9	1592	15.3	6268.0	40.5
Larotrectinib	4300.0	27.7	421.8	58.4	1745.0	16.5
Entrectinib	225.4	87.7	54.2	100.0	203.6	89.2
Selpercatinib (100 nM) +larotrectinb	10.0	100.0	54.5	93.9	49.6	61.4
Selpercatinib (100 nM) +entrectinib	1.8	100.0	14.2	100.0	8.5	100.0

^a^at 1000 nM of test drug concentration.

## Discussion

We report here that adaptive combination therapy can be used to improve treatment outcomes in patients with cancer who frequently exhibit molecular evolution of genetic profile. Additionally, we show the possibility of using liquid biopsy to guide treatment decisions and the practicality of using customized personalized custom combination therapies, also sometimes referred to as n-of-1 trial, to investigate possible newer combinations. N-of-1 trials are more patient-centered and allow for adaptation to evolving mechanisms of resistance throughout treatment^25,29,30^.

Three drugs were used in treatment of this patient including selpercatinib, larotrectinib, and entrectinib. Selpercatinib is a selective RET inhibitor that is currently FDA approved in patients with advanced medullary thyroid carcinoma and *RET* mutations and in *RET* fusion-driven thyroid cancer regardless of histology. Larotrectinib is NTRK inhibitor currently approved in patients with solid tumors who have *NTRK* fusions. Entrectinib is an inhibitor of NTRK, ALK, and ROS1 that is currently approved in patients with ROS1-positive non-small cell lung cancer and patients with solid tumors and *NTRK* gene fusions. Our preclinical data presented in Fig. 3 also suggest that entrectinib has a weak RET kinase inhibitor activity.

The baseline genetic profile suggested the presence of a previously uncharacterized four amino acid deletion (p.D898_E901del) in the RET kinase activation loop. Our experiment indicated that the p.D898_E901del mutation is a gain-of-function activating mutation, and has comparable sensitivity to selpercatinib. Consistently, the RET(D898_E901del)-positive patient had dramatic response to selpercatinib that persisted for two years. However, the tumors eventually developed resistance to RET inhibition via NTRK-driven mechanism, which has previously been described as a possible mechanism of resistance to selpercatinib.^23^ A trial for local therapy of progressing liver lesions was made but ultimately failed to control systemic disease, which necessitated change in management approach. Introduction of larotrectinib was made in a customized combination with selpercatinib which again led to disease control. This result validates our previously observation that suggested NTRK3 fusion is a targetable mechanism of acquired resistance to selpercatinib^23^. Nevertheless, emerging resistance occurred by developing an *EML4::ALK* fusion. To the best of our knowledge, ALK fusions have not been previously described as possible mechanism of resistance to either selpercatinib or larotrectinib. Secondary resistance to larotrectinib has been previously suggested to happen with NTRK new alterations (particularly in solvent front, gatekeeper, and xDFG positions), *MET* amplifications, *BRAF* p.V600E mutations, and *KRAS* mutations^31–33^. Given the dual ALK and NTRK inhibitory effect of entrectinib, a new combination was used to treat the patient by combined selpercatinib and entrectinib and led to prolonged disease control. Interestingly, despite stable scans, liquid biopsy suggested disease progression (in the form of increased VAF and new mutation in *NTRK3* p.G623R (previously reported as possible mechanism of resistance to larotrectinib^31^) which is consistent with increase in calcitonin levels. There is at least some evidence that progression observed in liquid biopsy generally precedes progression in imaging with a lead time^34,35^. This on-target *NTRK3* mutation is not covered by the currently FDA approved drugs larotrectinib and entrectinib and is a reported acquired mechanism of resistance. Repotrectinib and teletrectinib are two newer agents that are in development^36^. In order to intercept further progression a combination of RET + ALK + NTRK inhibitor that covers *NTRK3* p.G623R may be warranted in the future studies.

In the context of cancer treatment, the emergence of drug-resistant mutations or alterations, such as the acquisition of additional fusions, leads to the survival and proliferation of these cells despite the presence of tyrosine kinase inhibitors. This phenomenon mirrors the “Darwinian principles of natural selection”, wherein cells harboring mutations that confer them with the ability to thrive and multiply in the face of the drug’s pressure are the ones that endure and eventually give rise to a resistant tumor. This Darwinian selection process, specifically in relation to tyrosine kinase drug resistance and the development of acquired fusions, mirrors the evolutionary mechanisms through which cancer cells bearing particular genetic alterations that grant them a competitive advantage in the presence of targeted therapies gain dominance within a tumor population. Understanding and recognizing this dynamic is of paramount importance for the formulation of novel strategies like N-of-1 customized precision oncology therapies aimed at overcoming drug resistance and ultimately enhancing the outcomes of cancer treatment such as this case.

This case report marks the first instance of a patient diagnosed with RET aberrant cancer exhibiting both NTRK and ALK fusions, who underwent treatment with an NTRK inhibitor and subsequently with an NTRK/ALK inhibitor. It’s important to note that we previously documented a case involving a patient who developed an acquired KHDRBS1-NTRK3 fusion (K8;N14) following selpercatinib treatment in a KIF5B-RET fusion (K15;R12) positive lung cancer^23^. However, in that instance, the patient did not receive a combination NTRK and RET inhibitor. Furthermore, we have also incorporated and cited references to acquired fusions such as BRAF fusions and MET fusions, as they have been reported as resistance mechanisms to other driver alterations like EGFR^37–40^.

This case demonstrates the complexity of cancer evolution in RET-targeted therapy and the possibility of addressing such complexity using customized combination therapies that prolong tumor control. It also illustrates the utility of using liquid biopsy to guide timely decisions of personalized treatments (i.e. n-of-1 trials)^25^.

## Methods

### Immune complex kinase assay

The cDNA encoding flag-tagged wildtype RET and RET(D898_E901del) were cloned into pCDNA3.1. Plasmids of pCDNA3.1 vector, pCDNA3.1-RET, and pCDNA3.1-RET(D898_E901del) were transfected into HEK293 cells using lipofectamine-3000 based on supplier’s protocol. Two days after transfection, cells were lysed in Buffer A (50 mM Tris-HCl, pH7.5, 150 mM NaCl, 1 mM EDTA, 1 mM EGTA, 25 mM NaF, 5 mM Na_4_P_2_O_7_, 1 mM dithothreitol, 1 mM Na_3_VO_4_, 100 µg/ml phenylmethylsulfonyl fluoride, 2 µg/ml leupeptin, 2 µg/ml aprotinin, and 1% Triton X-100). Equal amounts of cleared cell lysates were immunoprecipitated with Pierce anti-DYKDDDK magnetic-agarose (ThermoFisher cat. No. A36797). The protein-bound beads were washed 4 times with the Buffer A and once with the Kinase Reaction Buffer (10 mM Tris-HCl, pH7.4, 50 mM NaCl, 10 mM MgCl_2_, 1 mM dithiothreitol, 10 µM ATP). The kinase reaction mixture contained the immune complex and 5 µg GST-GabCT protein in the Kinase Reaction Buffer.^40^ The kinase reaction was 8 min at 30 °C. The reaction was stopped by adding 4 x SDS-gel loading buffer and heated. Equal amounts of the supernatants were analyzed by immunoblotting with indicated antibodies.

### Transformation assay

The cDNA encoding flag-tagged wildtype *RET* and *RET*(D898_E901del) were cloned into a bicistronic lentiviral vector pWPI (Addgene plasmid #12254). Lentivirus were prepared in HEK293T cells and used to infect BaF3 cells cultured in RPMI-1640/10%FBS with 1 nM interleukin-3 (IL-3). One week after infection, GFP^+^ cells of similar GFP intensity and scattering level were sorted (1 cell/well) into 96-well plates in IL-3 free medium. Cell colonies that were able to grow in IL-3-free medium were manually countered under a microscope by examination of individual well 9 days after the cultured in the IL-3-free medium. Statistical analysis was performed using student’s unpaired *t*-test with Welch’s correction. p < 0.05 was considered statistically significant.

### Cell lines, IC_50_ determination, and immunoblotting assay

BaF3/RET(D898_E901del) cells were established similar to that of BaF3/RET(M918T) cells^17^. *ETV6::NTRK3* (E5;N14) coding cDNA was constructed from chemically synthesized DNA fragments (GenScript) and cloned into pGCXIN retrovirus vector. BaF3/RET(D898_E901del) cells infected with *ETV6::NTRK3* retrovirus were selected with puromycin and G418 and screen for expression of both the flag-tagged RET(D898_E901del) and *ETV6::NTRK3*. A hygromycin-resistant, HA-tagged *EML4::ALK* expression vector was constructed in retrovirus vector pQCXIH by cloning *EML4::ALK* variant 1 (E13;A30) from the pLenti-*EML4::ALK* variant 1 (E13;A30) plasmid obtained from Addgene (Plasmid #183828). BaF3/*EML4::ALK* cells were established as above for the RET oncogene gene cells. The triple alteration BaF3/RET(D898_E901del)/*ETV6::NTRK3*/*EML4::ALK* cells were established by infecting BaF3/RET(D898_E901del)/*ETV6::NTRK3* cells with lentivirus expressing *EML4::ALK*, selecting puromycin/G418/hygromycin-resistant cells in semi-solid MethoCult H4100 methylcellulose culture, and screening by immunoblotting for expression of Flag-tagged RET(D898_E901del), *ETV6::NTRK3*, and HA-tagged *EML4::ALK* proteins. Experiments using mixture of cells were performed by mixing an equal number of BaF3/RET(D898_E901del)/*ETV6::NTRK3* cells and BaF3/*EML4::ALK* cells. Selpercatinib IC_50_ for BaF3/RET(898_E901del) and BaF3/RET(M918T) cells were determined in parallel in the same experiments as decribed^17^. IC_50_ determinations of selpercatinib, larotrectinib, entrectinib, and their drug combinations in different cells were performed in parallel experiments. IC_50_ data were from at least two independent experiments performed in triplicates. Immunoblotting analyses were performed as described^17^. Antibodies used are listed in Supplementary Table s1.

### Clinical data

In reporting clinical data, we have complied with all relevant ethical regulations including the Declaration of Helsinki. The patient was treated as part of a clinical trial that was approved by the institutional review board at The University of Texas MD Anderson Cancer Center. Additional therapy beyond the clinical trial was personalized for the patient after informed consent and discussing benefits, risks, and side effects. Informed consent was obtained from the patient prior to starting any study-related procedures and for any publication from the study. Tissue DNA sequencing was performed using an inhouse MD Anderson paired tumor-normal next-generation sequencing assay that analyzed 146 cancer-related genes. Plasma sequencing was performed using MD Anderson cell-free DNA liquid biopsy NGS assay that analyzed 70 cancer-related genes. Patient permission to deposit raw sequencing data was not obtained separately and therefore the raw sequencing data could not be deposited.

### Reporting summary

Further information on research design is available in the Nature Research Reporting Summary linked to this article.

### Supplementary information

Reporting Summary

Supplementary Material

## Data Availability

Data reported in this article will be available upon reasonable request from corresponding authors.
