# Supplementary material for: Adaptive Darwinian off-target resistance mechanisms to selective RET inhibition in RET driven cancer
Source: NPJ Precis Oncol. 2024 Mar 4;8:62. doi: 10.1038/s41698-024-00563-4 (PMC10912412; doi:10.1038/s41698-024-00563-4)
Supplement: Supplementary file 2 — Supplementary Material [file 41698_2024_563_MOESM2_ESM.docx]

**Supplementary MATERIALS**

**Acquired *NTRK3* fusion, *ALK* Fusion, *NTRK3* solvent-front mutation as secondary, tertiary, and quaternary resistance mechanisms in *RET* mutant medullary thyroid carcinoma: Darwinian selection in action**

Vivek Subbiah^1^, Mohamed A. Gouda^1^, J. Bryan Iorgulescu^2^, Ramona Dadu^3^, Keyur Patel^2^, Steven Sherman^3^, Maria Cabanillas^3^, Mimi Hu^3^, Luz E. Castellanos^3^, Behrang Amini^1^, Funda Meric-Bernstam^1^, Tao Shen^4^, and Jie Wu^4^

^1^Department of Investigational Cancer Therapeutics, The University of Texas MD Anderson Cancer Center. Houston, Texas.

^2^Molecular Diagnostics Laboratory, Department of Hematopathology, Division of Pathology and Laboratory Medicine, The University of Texas MD Anderson Cancer Center. Houston, Texas

^3^Department of Endocrine Neoplasia, The University of Texas MD Anderson Cancer Center. Houston, Texas

^4^Peggy and Charles Stephenson Cancer Center and Department of Pathology, University of Oklahoma Health Sciences Center, Oklahoma City, Oklahoma

Correspondence to: Dr. Vivek Subbiah, Sarah Cannon Research Institute, 1100 Dr. Martin L. King Jr. Blvd, Suite 800, Nashville, TN 37203, USA; Tel. +1-629-900-5888, email: [Vivek.Subbiah@scri.com](mailto:Vivek.Subbiah@scri.com); or Dr. Jie Wu, University of Oklahoma Health Sciences Center, 975 NE 10^th^ Street, BRC 413, Oklahoma City, OK 73104, USA. Tel. +1-405-271-8001 ext. 31092; email: [jie-wu@ouhsc.edu](mailto:jie-wu@ouhsc.edu)

**Supplementary Table s1. List of antibodies**

| **Antibody name** | **Target** | **Dilution** | **Source** | **Catalog number** |
| --- | --- | --- | --- | --- |
| Phospho-RET(Y905) | RET pY905 | 1:1000 | CST* | 3221 |
| Phospho-NTRK1/2/3 | NTRKC (pY709/710) | 1:500 | CST | 4621 |
| Phospho-ALK (Y1507) | ALK pY1507 | 1:1000 | CST | 14678 |
| Phospho-ALK (Y1604) | ALK pY1604 | 1:1000 | CST | 3341 |
| Flag tag | Flag tag | 1:1000 | Sigma | F1804 |
| HA tag | HA tag | 1:000 | CST | 3724 |
| ETV6 (TEL) | ETV6 | 1:500 | Santa Cruz | SC166835 |
| Cleaved PARP | Cleaved PARP D214 | 1:1000 | CST | 9541 |
| ACTIN | β-ACTIN | 1:5000 | Sigma | A5441 |

*, Cell Signaling Technology


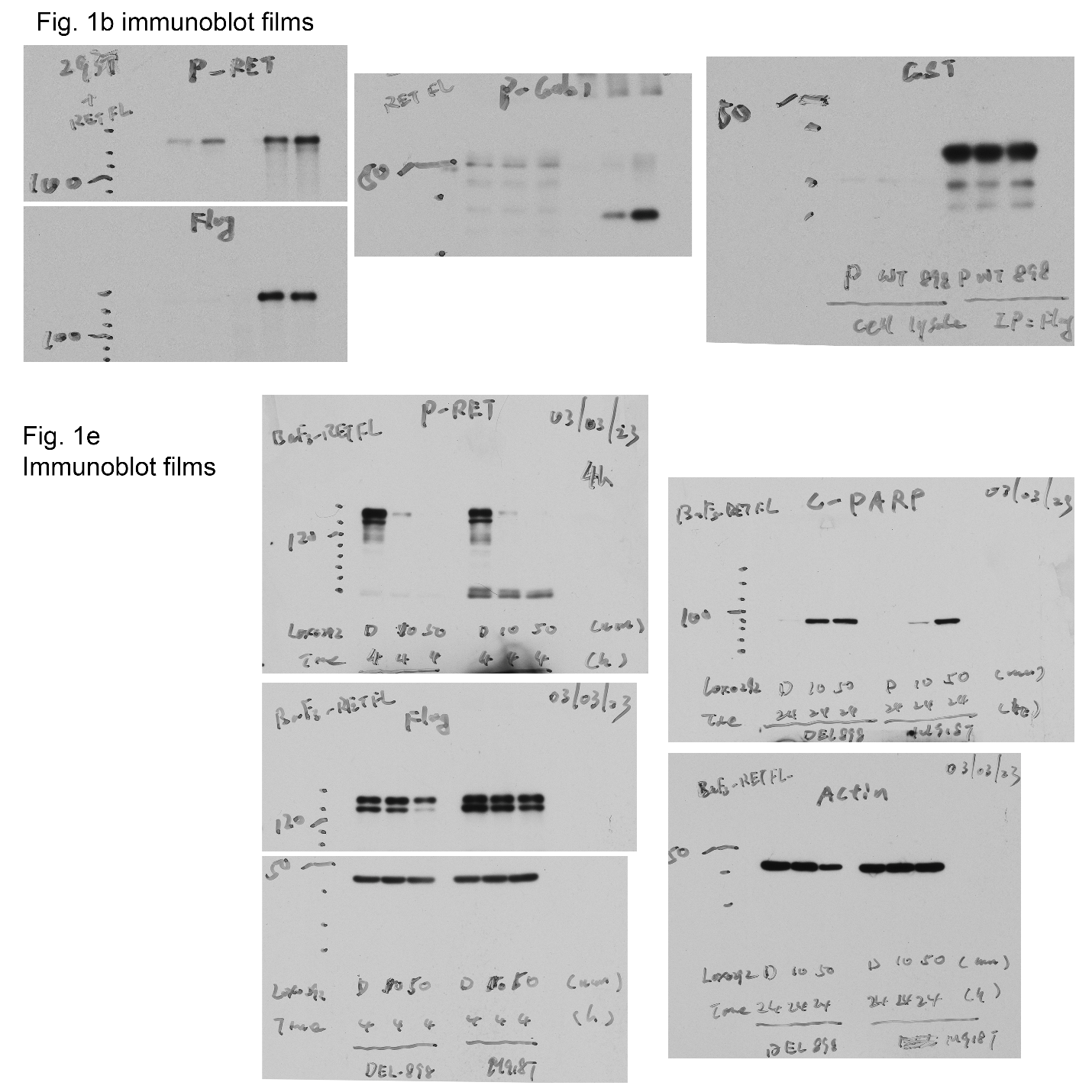


**Supplementary Fig. s1. Original immunoblot film scans used for Fig. 1b and 1e.**


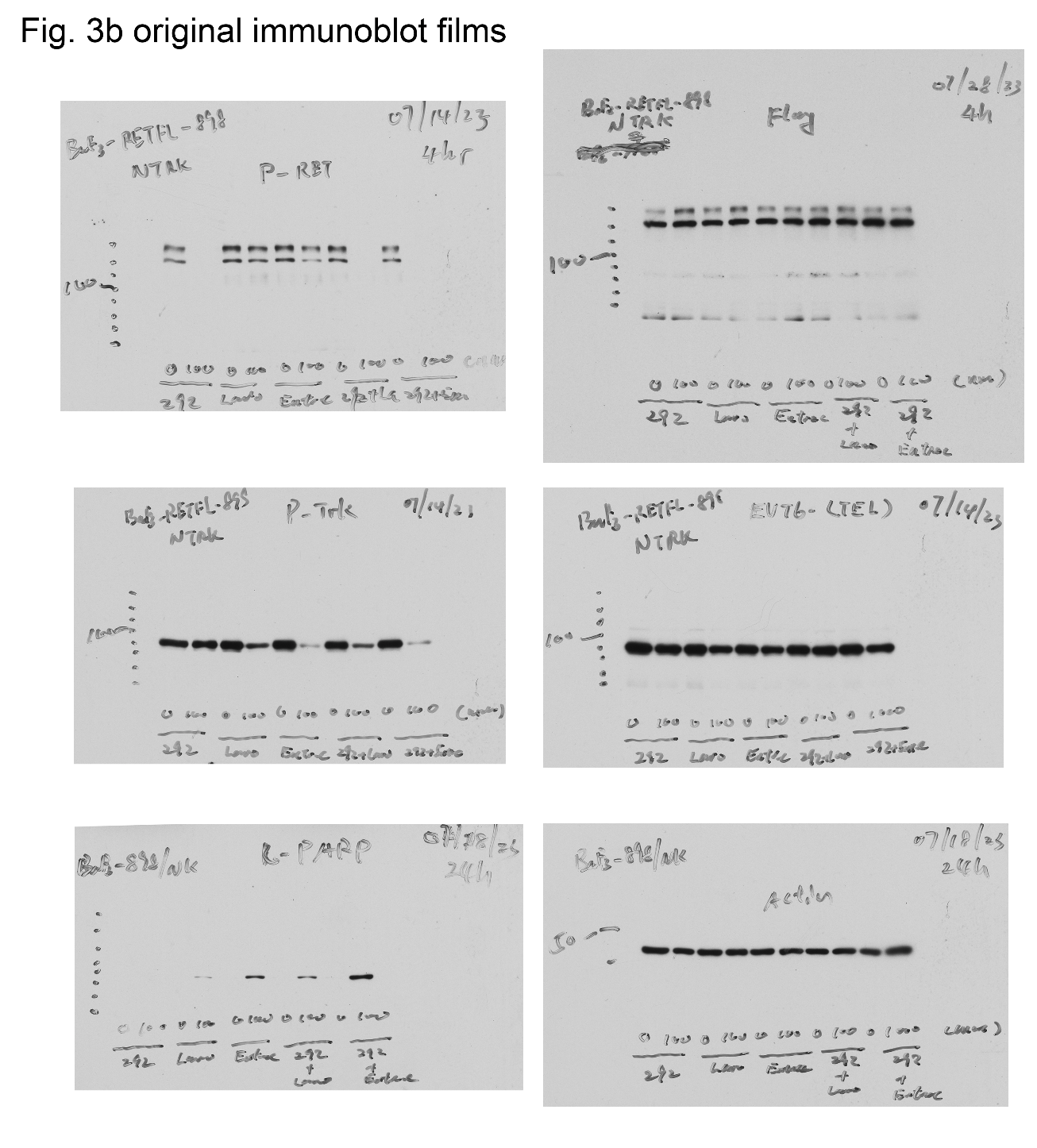


**Supplementary Fig. s2. Original immunoblot film scans used for Fig. 3b.**

**
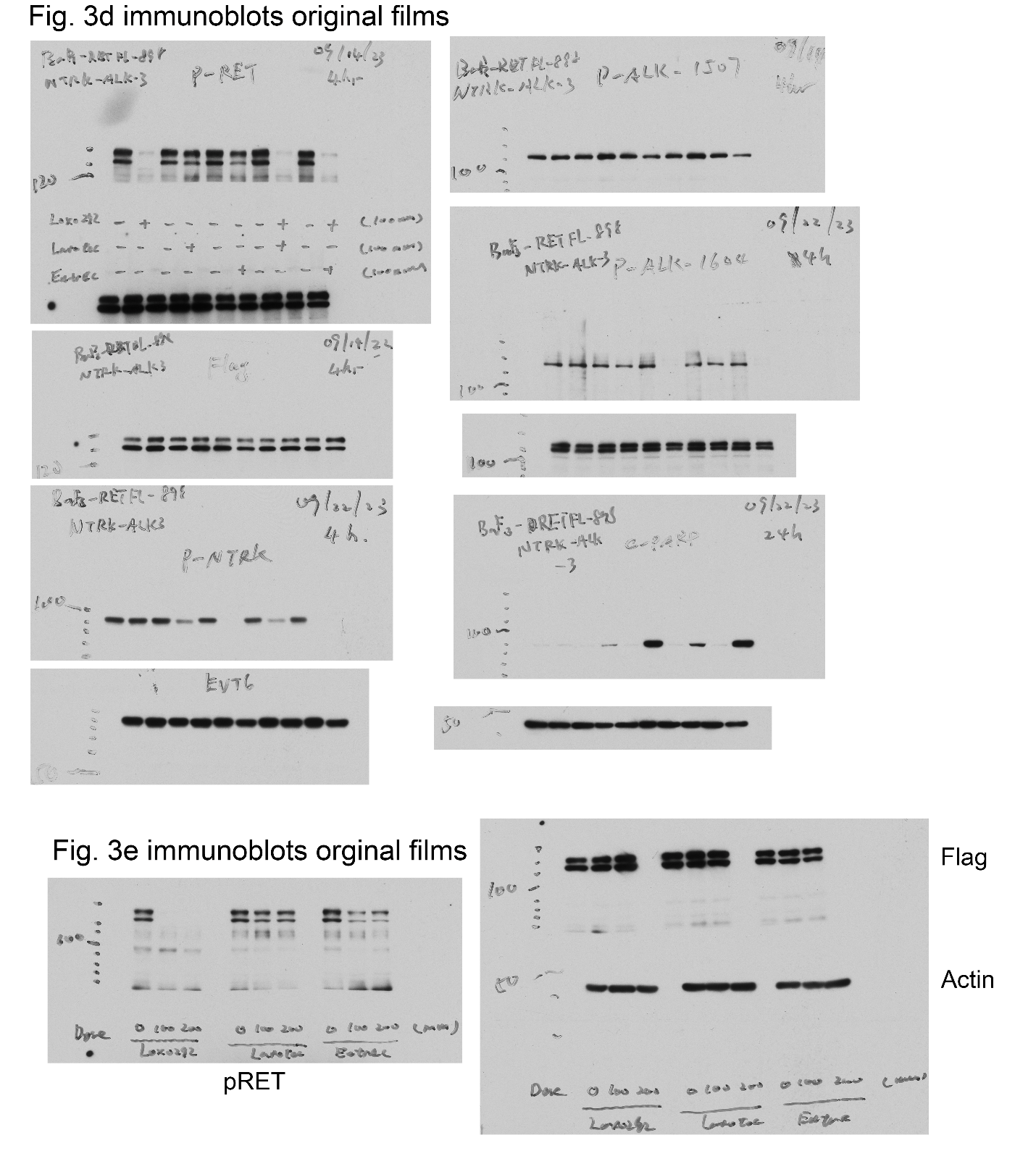
**

**Supplementary Fig. s3. Original immunoblot film scans used for Fig. 3d and 3e.**

**
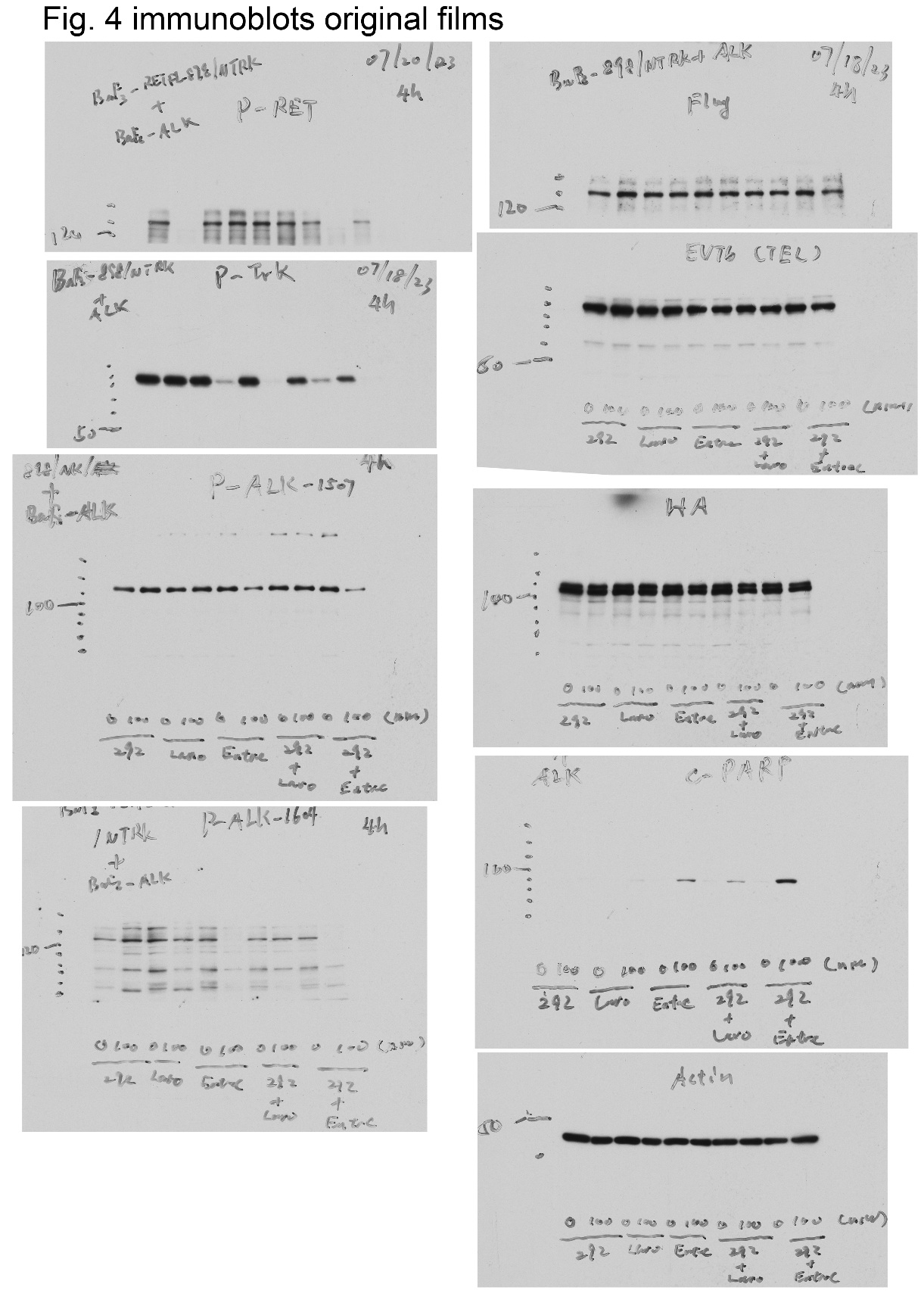
**

**Supplementary Fig. s4. Original immunoblot film scans used for Fig. 4b.**
